# An investigation into social determinants of health lifestyles of Canadians: a nationwide cross-sectional study on smoking, physical activity, and alcohol consumption

**DOI:** 10.1186/s12889-024-19427-4

**Published:** 2024-08-01

**Authors:** Xiangnan Chai, Yongzhen Tan, Yanfei Dong

**Affiliations:** 1 Department of Sociology, School of Social and Behavioral Sciences, Nanjing University, Nanjing, Jiangsu P. R. China; 2 Law School, Shihezi University, Shihezi, Xinjiang P. R. China; 3 Department of Social Work and Social Policy, School of Social and Behavioral Sciences, Nanjing University, Nanjing, Jiangsu P. R. China

**Keywords:** Social determinants of health, Health lifestyles, Canadian adults, Cigarette smoking, Physical activity, Alcohol drinking, Health region of Canada

## Abstract

**Background:**

Health lifestyles exert a substantial influence on the quality of everyday life, primarily affecting health maintenance and enhancement. While health-related practices during the COVID-19 pandemic may have positively altered the health lifestyles of Canadians to a certain degree, government reports indicate that issues related to health behaviors, such as cigarette smoking, physical inactivity, and alcohol consumption, continue to pose challenges to the health of Canadians. Social determinants of these health behaviors thus hold significant academic value in the formulation of policy guidelines.

**Objective:**

The aim of this study is to scrutinize the social determinants of health with respect to social factors that have may have impacts on the health-related behaviors of Canadians. We tested health behaviors including cigarette use, alcohol consumption, and participation in physical exercise, which are integral to the promotion and improvement of individual health.

**Methods:**

To examine the social determinants of Canadians’ health lifestyles, we utilized nationally representative data from the 2017–2018 Canadian Community Health Survey annual component. Our data analysis involved the bootstrapping method with two-level mixed-effect logistic regressions, ordered logistic regressions, and negative binomial regressions. Additionally, we conducted several robustness checks to confirm the validity of our findings.

**Results:**

The findings show that demographic background, socioeconomic status, social connections, and physical and mental health conditions all play a role in Canadians’ smoking, physical activity, and drinking behaviors. Noticeably, the association patterns linking to these social determinants vary across specific health lifestyles, shedding light on the complex nature of the social determinants that may influence young and middle-aged Canadians’ health lifestyles. Moreover, in the context of Canada, the health-region level demographic, socioeconomic, and working conditions are significantly linked to residents’ health lifestyles.

**Conclusions:**

Investigating the social determinants of health lifestyles is pivotal for policymakers, providing them with the necessary insights to create effective interventions that promote healthy behaviors among specific demographic groups. It is recommended that health education and interventions at the community level targeting smoking, physical inactivity, and alcohol consumption be introduced. These interventions should be tailored to specific subgroups, considering their demographic and socioeconomic characteristics, social networks, and health status. For instance, it is imperative to focus our attention on individuals with lower educational attainment and socioeconomic status, particularly in relation to their smoking habits and physical inactivity. Conversely, interventions aimed at addressing alcohol consumption should be targeted towards individuals of a higher socioeconomic status. This nuanced approach allows for a more effective and tailored intervention strategy.

## Background

Researchers from different disciplines have shown increasing interest in health lifestyles as a key topic in health studies [1, 2]. Previous studies have shown that health lifestyles consist of various aspects, such as tobacco use, alcohol consumption, food choices, substance abuse, exercise, and sleep behavior and quality [3–5]. Since health lifestyles are closely related to people’s everyday life, they have a major impact on health preservation and health enhancement. A WHO report [6] states that human behavior and lifestyle factors are significant factors that affect an individual’s health. Studies across countries reveal that unhealthy lifestyles predispose to hypertension [7], diabetes [8], cardiovascular disease [9], kidney [10, 11], fatty liver [12], lung cancer [5], heart disease [13], multiple sclerosis [14], breast cancer [15], and some other diseases. Mental health can also be significantly influenced by health lifestyles. Rohrer et al. [16] conducted a social survey among residents of Amarillo, Texas, and discovered that unhealthy lifestyles, such as daily smoking, and their consequences, such as obesity, are related to poor mental health. Therefore, intervention is important. According to Walsh [17], patients’ mental health status can be effectively improved by lifestyle changes in a therapeutic context. Similarly, Dale et al. [18] found in a systematic literature review that positive changes in health behaviors, such as physical exercise, diet, and smoking, can effectively enhance people’s mental well-being.

This study examined the effects of micro- and meso-level variables on the health behaviors of Canadians, including cigarette smoking, physical activity, and alcohol drinking. These variables were chosen based on the framework emphasizing social determinants of health lifestyles. The results have important implications for policy interventions that aim to improve the health of Canadians.

### Social determinants of health lifestyles

Health lifestyles can be understood as “collective patterns of health-related behavior based on choices from options available to people according to their life chances.” [19]. The health lifestyle theory, proposed by Cockerham and colleagues [19, 20], posits that people’s health lifestyles are shaped by the interaction between their life choices (human agency) and life chances (social structure), which form their habitus (Bourdieu’s term for dispositions to act) and subsequent daily actions. The factors that affect people’s life chances and choices are the social determinants of health lifestyles, such as demographic characteristics (e.g., age, gender, ethnicity) and socioeconomic status (social class, living conditions, etc.). This suggests that health lifestyles are not arbitrary or independent health behaviors, but rather are conditioned and influenced by the structural factors that individuals encounter. Empirical studies have addressed factors at the micro-, meso-, and macro-levels as underlying contributors that may affect people’s health lifestyles. Demographic backgrounds, such as gender, race/ethnicity, and age are closely associated with health lifestyles [3, 20, 21]. Cockerham and colleagues [22–24] have argued that Indicators of socioeconomic status, including income, occupation, and educational levels, have been argued as protective factors in this vein. Besides, residential environment regarding household and neighborhood quality has a significant impact on people’s choices regarding health behaviors and lifestyles [25]. According to a recent empirical study, children’s sedentary behaviors can be effectively reduced by the walkability of neighborhood, and this positive effect can be further enhanced by favorable parenting practices in physical activity [26]. On top of that, sociocultural contexts, such as different food and cultural practices and the political ideologies across different countries have either positive or negative influences on how people would develop their health lifestyles [3, 27]. These observations suggest that systematic factors play into one’s capacity to adjust to lifestyle interventions and effectuate behavioral modifications. In other words, while health lifestyles are intimately intertwined with individuals’ daily routines, they are profoundly influenced by structural determinants.

Furthermore, the occurrence of lifestyle stigmatization is widespread across diverse societies. Current scholarly works suggest the presence of societal pressures linked to the stigmatization of health-oriented lifestyles and their outcomes, such as overweight and obesity [28]. Consequently, the internalization of lifestyle stigmatization could potentially impact an individual’s ability to embrace healthier lifestyles and instigate behavioral changes, such as reducing excessive smoking and drinking, and increasing physical activity. Nonetheless, structural elements like educational achievements, income levels, and living arrangements may play a more pivotal role in shaping health behaviors. The exploration of these influences constitutes the focus of our present study.

### The Canadian context: unhealthy lifestyles facing Canadian adults

Governmental reports show that Canadians have some unhealthy lifestyle habits, such as smoking and drinking heavily, and being physically inactive. For example, recent Canadian surveys reveal that almost 18% of people aged 12 and over smoke daily or occasionally [29]. Although the number of Canadian smokers has been declining steadily, the current percentage is still high and concerning. In terms of physical activity, in 2012, nearly 77.8% of Canadian adults aged 18 and above did not meet national physical activity guidelines [29]. Although the percentage has decreased to 47.0% in 2020, being physical inactive is still an issue facing many Canadians [30]. Regarding drinking, in 2013, about 16.0% of Canadians aged 15 and over exceeded national drinking guidelines; the age-standardized percentage of Canadians who had heavy drinking problems reached 17.9% in 2014 [29]. Despite the COVID-19 pandemic may have changed Canadians’ health lifestyles to some extent, an official report indicates that cigarette smoking, physical inactivity, alcohol drinking, and some other health behavior problems, such as substandard fruit and vegetable intake, still challenging Canadians’ health [31]. These figures indicate that many Canadians have unhealthy lifestyle habits. However, there is a lack of systematic investigation on the factors that relate to Canadians’ health lifestyles.

### The current research

The objective of this research is to explore the social determinants that influence the health behaviors of Canadians, encompassing habits such as cigarette consumption, alcohol intake, and engagement in physical activities. These behaviors play a pivotal role in promoting and enhancing an individual’s health. Our analysis delves into the social determinants that shape the health behaviors of Canadians at various levels - individual, household, and regional.

## Methods

### Data

Our data source is the Canadian Community Health Survey (CCHS) Annual Component. CCHS, a series of repeated cross-sectional datasets that capture the physical and mental health conditions of Canadians aged 12 and above, was collected by Statistics Canada. We employed the 2017–2018 CCHS to investigate the social determinants of health lifestyles. The 2017–2018 CCHS included information on Canadians’ health behaviors regarding cigarette smoking, alcohol drinking, and doing physical exercises, as well as on Canadians’ demographic characteristics, socioeconomic status, and social connections, which allows us to conduct robust modelling in testing social determinants of health behaviors. The nationally representative feature of the data ensures the validity and applicability of our findings.

### Analytical sample

This study targets Canadian adults aged above 18 and under 60, namely young and middle-aged Canadians. The initial sample size is 62,061. We remove respondents with missing data in any of the outcome variables and some of the independent variables, which occupies 3.64% of the original sample. The final sample size is 59,799. The exclusion of missing cases was justified by their relatively minor proportion. Furthermore, we have categorized missing cases in independent variables as a distinct group, “missing”. The analytical outcomes, which are consistent with our presented results, suggest that the exclusion of cases with missing values does not lead to biased estimations. Moreover, CCHS utilizes a multi-stage sample allocation strategy and provides a weighting variable within its dataset for analysis.

### Measures

#### Dependent variables

We test health behaviors variables related to smoking, drinking, and physical exercise as dependent variables. For smoking, we include four variables. “Lifetime cigarette smoking” was assessed with “Have you ever in your life smoked a whole cigarette (no/yes)?”, “Lifetime consumption of more than 100 cigarettes” was measured based on the survey question “Have you smoked more than 100 cigarettes (about 4 packs) in your life (no/yes)?”, “Current smoking status” was measured with “At the present time, do you smoke cigarettes every day (daily), occasionally (occasional) or not at all (none)?”, and “Quitting smoking” was based on “When did you stop smoking daily?” and was coded into two categories, “Current occasional/daily smoker” and “completely quitted former smoker.”

As for drinking, we included two variables: “Current drinking status” was coded using the question “Type of drinker [in the past] 12 months (None, occasional, regular,” and “Frequency of alcohol consumption in the past 12 months” was assessed with “During the past 12 months, how often did you drink alcoholic beverages (No alcohol, < 1/month, 1/month, 2–3/month, 1/week, 2–3/week, 4–6/week, and daily?” Finally, we evaluated how well each respondent met the Canadian Physical Activity Guidelines for physical activity (CPAG), which classifies Canadians’ physical activity level into three categories: at/above recommended level from CPAG, below recommended level from CPAG, and minimal physical activity reported. We also examined two other comparable variables, “Physically active based on WHO guidelines (level 1 to level 4)” and “Alternate physical activity indicator (level 1 to level 4)” to ensure the robustness of our results. For both variables, level 1 indicates the highest level of being physical active and level 4 indicates the lowest.

### Independent variables

We use the following variables to measure different aspects of personal characteristics. The variables under consideration are examined for their predictive capacity in relation to the social determinants of health behaviors within the adult population of Canada. We include two levels of predictors with the first level of respondents’ characteristics, and the second level variable is on health region of Canada.

Four types of variables are included as the first-level predictors, including people’s demographic backgrounds, socioeconomic status, social connections, and health conditions. we include four First, demographic backgrounds: age group (10-year intervals), sex (female, male), race (non-minority, minority, missing), immigration status (Canadian born, landed immigrant, missing), and province of residence (Ontario, Quebec, British Columbia, Alberta, Other provinces and territories). Second, socioeconomic status (SES): education level (primary or below, middle/high school, some college or above, missing), household income (< 20 K, 20–39.9 K, 40–59.9 K, 60–79.9 K, 80 K + Canadian dollars), and dwelling ownership (owned, rented, missing). Third, social connections: marital status and living arrangements combined (married not alone, married alone, common-law not alone, common-law alone, widowed/divorced/separated not alone, widowed/divorced/separated alone, single not alone, single alone), and sense of community belonging. The sense of belonging to local community is based on the survey question “How would you describe your sense of belonging to your local community? Would you say it is. (very weak, somewhat weak, somewhat strong, very strong, missing)?” Noticeably, due to data limitation, we were not able to use other social connection variables that may affect health outcomes, such as how often Canadians meet or contact their friends or relatives [32]. Lastly, health conditions: chronic disease (no, yes), mood issues (no, yes), and anxiety issues (no, yes), and whether having a regular health provider (no, yes).

On top of above-tested first-tier independent variables, we also include a second-tier variable at the health region level. We opted to utilize a health-region-level variable: the health region peer group variable. This variable, provided by Statistics Canada, is preferred over the direct use of the health region variable offered by CCHS. The rationale behind this choice is that the health-region-peer-group variable incorporates demographic and socioeconomic conditions as well as working environments of health regions, as per Statistics Canada. In essence, examining variations in health lifestyles over this variable allows us to understand how regional level conditions may have influenced the health lifestyles of Canadians, which aligns with our application of hierarchical mixed-effect models.

### Modelling technique

We used two-level mixed-effect binary and ordinal logistic regressions and negative binominal regressions to test outcomes that are coded as dichotomous, ordinal, or count variables. Given the sampling design of the CCHS, Canadian respondents are grouped within various health regions. It is plausible that individuals from the same health region peer group may exhibit similar health lifestyles. Consequently, the health region peer group can be conceptualized as a cluster, representing the random effect in our model. We conducted tests for multicollinearity, and the findings indicate that there are no relevant issues. We also conducted a bunch of robustness tests to strengthen the validity of our results. Specifically, we have generated a new variable on alcohol drinking frequency, and applied other related regression models to predict our dependent variables. The results are in consistency with what we present.

### Ethical considerations

This research uses secondary data that was gathered and published by Statistics Canada. The data is publicly available and can be obtained from the Internet. No ethical issues arose from this research as it did not involve any human or animal experiments. More information on the data collection can be accessed from the following website: https://www23.statcan.gc.ca.

### Findings

#### Weighted characteristics of the analytical sample

Table 1 shows the weighted percentages of the dependent variables. It indicates that 53.12% of the respondents have smoked a whole cigarette before, and 37.78% have smoked one hundred cigarettes in their lifetime. There are 12.94% of respondents who smoke daily, and 6.01% who smoke occasionally. Among those who never smoke, have smoked, or still smoke, 34.17% have quit smoking completely. Regarding drinking, more than 65% of Canadians are regular drinkers, while 16.01% drink occasionally. Although 61.42% of Canadians meet the standards of Canadian Physical Activity Guidelines for physical activity, 23.14% of them are below the recommended level and 15.44% of them do minimal physical activity in daily life.

**Table 1:** Weighted percentages of the dependent variables, Canadian Community Health Survey 2017–2018. *N* = 59,799

Dependent variables	%	95% CIs
***Lifetime smoking (A whole cigarette)*** – Yes	53.12%	52.42-53.82%
***Lifetime smoking (100 cigarettes)*** – Yes	37.78%	37.12-38.44%
***Quitted smoking*** – Completely quitted	34.17%	33.53-34.82%
***Current smoking status***		
None	81.05%	80.53-81.56%
Occasionally	6.01%	5.69-6.34%
Daily	12.94%	12.52-13.38%
***Current drinking status***		
None	17.58%	17.00-18.18%
Occasional	16.01%	15.51-16.53%
Regular	66.41%	65.72-67.09%
***Physical inactivity***		
Above recommended level	61.42%	60.74-62.10%
Below recommended level	23.14%	22.56-23.73%
Minimal physical activity	15.44%	14.93-15.96%

**Fig. 1:**
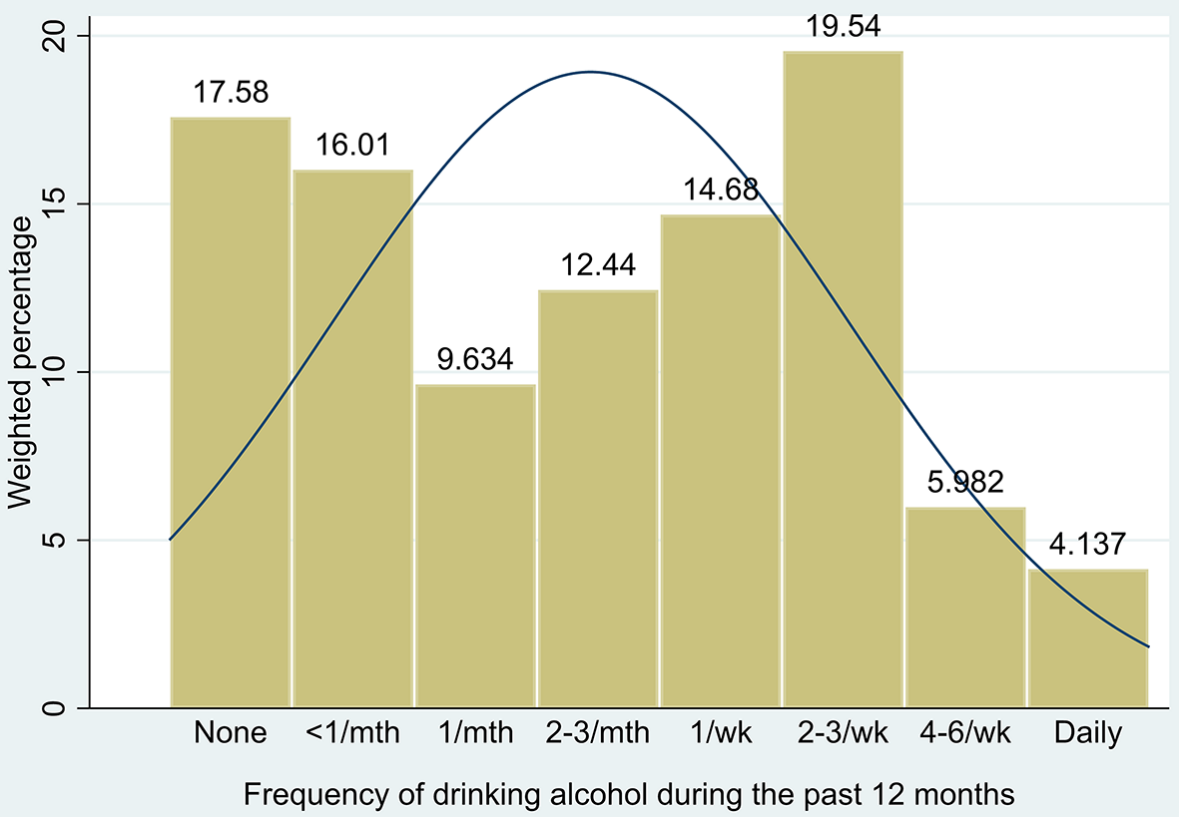
Weighted percentages of each category of the alcohol drinking frequency variable

We plotted the alcohol drinking frequency variable separately because it can be seen as a count variable. Figure 1 shows the weighted percentages of the value of each category. The lowest percentage (17.58%) is for respondents who did not drink at all in the past year, followed by those who drank less than once per month (16.01%). The percentage increases from once per month (9.63%) to once per week (14.68%). The most common drinking frequency among Canadians aged 18–60 is two to three times per week (19.54%). A relatively small proportion of respondents reported drinking more often, with 5.98% drinking four to six times per week and 4.14% drinking daily. The density distribution curve shows that the respondents are not normally distributed.

Table 2 presents the weighted percentages for all independent variables. The data shows a balanced distribution of respondents across age groups and genders. A certain percentage of respondents identify as minorities or landed immigrants, reflecting Canada’s diverse racial and immigration backgrounds.

**Table 2:** Weighted percentages of the independent variables, Canadian Community Health Survey 2017–2018. *N* = 59,799

Independent variables			
***Demographic background***		80k+	58.69%
**Age groups**		**Household ownership**	
18–29 years	27.07%	Rent	29.74%
30–39 years	25.24%	Owned	69.44%
40–49 years	22.95%	Missing	0.82%
50–59 years	24.74%	**Has healthcare provider**	
**Gender**		No	18.75%
Male	49.96%	Yes	81.25%
Female	50.04%	***Social connections***	
**Race**		**Marital status and living arrangements**	
Non-minorities	68.18%	Partnered, not living alone	45.09%
Minorities	30.59%	Partnered, living alone	0.54%
Missing	1.23%	Common-law, not living alone	15.40%
**Immigrant status**		Common-law, living alone	0.14%
Native born	71.28%	Widowed/Divorced/Separated, not living alone	4.17%
Landed immigrant	27.55%	Widowed/Divorced/Separated, living alone	2.89%
Missing	1.16%	Single, not living alone	23.29%
**Province**		Single, living alone	8.48%
Ontario	38.98%	**Sense of belongingness**	
Quebec	22.70%	Very weak	7.94%
British Columbia	13.02%	Somewhat weak	26.05%
Alberta	12.47%	Somewhat strong	49.31%
Others	12.82%	Very strong	14.46%
***Socioeconomic status***		Missing	2.24%
**Education**		***Health conditions***	
Primary or below	7.01%	**Chronic conditions**	
Middle/High school	24.45%	No chronic disease	73.70%
Some colleges or above	67.23%	Has at least one chronic disease	26.30%
Missing	1.31%	**Has mood issues**	
**Household income**		No	90.44%
< 20k	6.47%	Yes	9.56%
20k-39.9k	10.01%	**Has anxiety issues**	
40k-59.9k	12.65%	No	89.97%
60k-79.9k	12.18%	Yes	10.03%
		***Health region peer group***	
Health region peer group A	2.89%	Health region peer group E	1.74%
Health region peer group B	36.37%	Health region peer group F	0.18%
Health region peer group C	16.12%	Health region peer group G	16.90%
Health region peer group D	12.87%	Health region peer group H	12.94%

In terms of socioeconomic status, many Canadians have achieved a good level of education, have a stable household income, own their homes, and have access to a healthcare provider. However, it’s important to note that a significant portion of Canadians aged 18–60 are socioeconomically disadvantaged.

Nearly half of the respondents are in a partnership and not living alone, likely with their partners and/or children. Some are in common-law relationships and not living alone. A small percentage of those who are partnered or cohabiting live alone. Many Canadians are widowed, divorced, or separated. Some respondents are single but live with others (like parents or friends for the young, relatives or others for the middle-aged), and some singles live alone. More than half of the respondents feel they have somewhat strong to very strong ties with their communities. Health issues such as chronic diseases, mood disorders, and anxiety are also present among respondents.

At the provincial level, Ontario has the largest share of respondents and the highest GDP among all provinces and territories, followed by Quebec, Alberta, British Columbia, and so forth.

### Social determinants of smoking behaviors among Canadians

The relationships between smoking behaviors of Canadians aged 18–60 and four sets of independent variables are shown in Table 3. The results are consistent across models and reveal some demographic differences. Older respondents are more likely to smoke, regardless of the amount or frequency, than younger ones. Female and landed immigrant respondents have lower odds of smoking and higher odds of quitting than male and native respondents, respectively. However, racial minority respondents, while less likely to ever smoke, have higher odds of being occasional or daily smokers and lower odds of quitting than non-minority respondents. Geographic differences in cigarette smoking and quitting also exist among Canadians. Those residing in Quebec and British Columbia are less likely to smoke and more likely to quit if they do smoke, compared to those living in Ontario.

**Table 3:** Odds ratios from two-level binary logistic regressions predicting lifetime smoking and quitting smoking, and odds ratios from two-level ordinal logistic regressions predicting current smoking status, Canadian Community Health Survey 2017–2018, N of individual size = 59,799, N of health region peer group = 8

	Lifetime smoking (A whole cigarette)	Lifetime smoking (100 cigarettes)	Smoking status	Quitted smoking
***Demographic background***				
**Age groups (18–29 years)**				
30–39 years	2.03 (1.93, 2.14)***	2.54 (2.40, 2.69)***	1.67 (1.56, 1.78)***	0.91 (0.84, 0.98)*
40–49 years	2.38 (2.24, 2.51)***	3.06 (2.88, 3.25)***	1.67 (1.56, 1.79)***	1.02 (0.94, 1.11)
50–59 years	3.13 (2.95, 3.32)***	4.17 (3.91, 4.44)***	1.67 (1.55, 1.78)***	1.18 (1.09, 1.28)***
** Gender (Male)**				
Female	0.64 (0.62, 0.66)***	0.71 (0.68, 0.73)***	0.67 (0.65, 0.70)***	1.28 (1.22, 1.34)***
** Race (Non-minorities)**				
Minorities	0.79 (0.75, 0.83)***	0.90 (0.85, 0.94)***	1.08 (1.02, 1.15)**	0.80 (0.75, 0.85)***
Missing	0.79 (0.63, 0.98)*	0.87 (0.69, 1.10)	0.87 (0.67, 1.13)	1.05 (0.76, 1.45)
** Immigrant status (Native-born)**				
Landed immigrant	0.52 (0.49, 0.55)***	0.52 (0.49, 0.55)***	0.56 (0.52, 0.60)***	1.22 (1.12, 1.33)***
Missing	0.62 (0.52, 0.74)***	0.56 (0.47, 0.67)***	0.58 (0.46, 0.71)***	1.33 (1.03, 1.72)*
** Province (Ontario)**				
Quebec	1.04 (0.99, 1.10)	1.01 (0.96, 1.07)	0.84 (0.79, 0.89)***	1.26 (1.17, 1.35)***
British Columbia	1.03 (0.97, 1.09)	0.96 (0.90, 1.02)	0.74 (0.69, 0.80)***	1.47 (1.36, 1.60)***
Alberta	1.01 (0.94, 1.08)	0.99 (0.92, 1.06)	0.92 (0.85, 1.00)	1.12 (1.03, 1.22)*
Others	1.02 (0.97, 1.08)	1.00 (0.95, 1.05)	0.92 (0.86, 0.98)**	1.14 (1.06, 1.22)***
***Socioeconomic status***				
** Education (Primary or below)**				
Middle/High school	0.65 (0.60, 0.70)***	0.61 (0.57, 0.65)***	0.59 (0.55, 0.63)***	1.50 (1.39, 1.63)***
Some colleges or above	0.51 (0.48, 0.55)***	0.42 (0.39, 0.45)***	0.39 (0.37, 0.42)***	2.20 (2.05, 2.37)***
Missing	0.71 (0.59, 0.86)***	0.68 (0.57, 0.81)***	0.74 (0.61, 0.90)**	1.21 (0.96, 1.52)
** Household income (< 20k)**				
20k-39.9k	1.00 (0.92, 1.08)	0.99 (0.91, 1.08)	0.97 (0.90, 1.05)	1.08 (0.98, 1.18)
40k-59.9k	0.94 (0.87, 1.02)	0.86 (0.79, 0.93)***	0.82 (0.76, 0.89)***	1.28 (1.16, 1.41)***
60k-79.9k	0.91 (0.84, 0.99)*	0.84 (0.77, 0.91)***	0.75 (0.69, 0.82)***	1.39 (1.26, 1.54)***
80k+	0.83 (0.77, 0.89)***	0.68 (0.63, 0.73)***	0.58 (0.54, 0.62)***	1.77 (1.61, 1.94)***
**Household ownership (Rent)**				
Owned	0.75 (0.72, 0.79)***	0.72 (0.69, 0.75)***	0.65 (0.62, 0.68)***	1.39 (1.31, 1.47)***
Missing	1.15 (0.86, 1.54)	1.24 (0.92, 1.66)	0.99 (0.71, 1.37)	1.08 (0.72, 1.63)
***Social connections***				
**Marital status and living arrangements** **(Partnered**, **not living alone)**				
Partnered, living alone	0.87 (0.73, 1.03)	0.94 (0.78, 1.13)	1.13 (0.89, 1.42)	0.78 (0.61, 1.00)
Common-law, not living alone	1.58 (1.49, 1.67)***	1.75 (1.65, 1.85)***	1.97 (1.85, 2.11)***	0.59 (0.55, 0.63)***
Common-law, living alone	1.36 (0.97, 1.91)	1.17 (0.86, 1.61)	1.93 (1.38, 2.70)***	0.53 (0.36, 0.77)**
Widowed/Divorced/Separated, not living alone	1.29 (1.19, 1.40)***	1.35 (1.25, 1.46)***	1.77 (1.61, 1.95)***	0.59 (0.53, 0.65)***
Widowed/Divorced/Separated, living alone	1.47 (1.36, 1.60)***	1.51 (1.39, 1.63)***	2.04 (1.87, 2.22)***	0.52 (0.47, 0.57)***
Single, not living alone	1.09 (1.03, 1.16)**	1.15 (1.08, 1.22)***	1.86 (1.74, 2.00)***	0.46 (0.43, 0.50)***
Single, living alone	1.12 (1.06, 1.19)***	1.10 (1.04, 1.17)**	1.56 (1.45, 1.67)***	0.60 (0.56, 0.65)***
**Sense of belongingness (Very weak)**				
Somewhat weak	0.99 (0.92, 1.07)	0.93 (0.87, 1.00)	0.88 (0.81, 0.95)**	1.18 (1.07, 1.29)***
Somewhat strong	0.91 (0.85, 0.98)*	0.86 (0.80, 0.92)***	0.85 (0.79, 0.92)***	1.17 (1.08, 1.28)***
Very strong	0.84 (0.77, 0.91)***	0.82 (0.76, 0.88)***	0.89 (0.81, 0.97)**	1.03 (0.93, 1.13)
Missing	0.46 (0.40, 0.53)***	0.50 (0.43, 0.59)***	0.57 (0.48, 0.69)***	1.03 (0.84, 1.27)
***Health conditions***				
**Chronic conditions**				
Has at least one chronic disease	1.12 (1.07, 1.16)***	1.18 (1.13, 1.22)***	1.09 (1.04, 1.14)***	0.97 (0.92, 1.02)
**Has mood issues (No)**				
Yes	1.40 (1.31, 1.49)***	1.40 (1.32, 1.50)***	1.43 (1.34, 1.53)***	0.78 (0.73, 0.84)***
**Has anxiety issues (No)**				
Yes	1.46 (1.37, 1.56)***	1.52 (1.42, 1.62)***	1.51 (1.41, 1.62)***	0.74 (0.69, 0.80)***
**Has healthcare provider (No)**				
Yes	0.85 (0.81, 0.89)***	0.84 (0.80, 0.87)***	0.71 (0.67, 0.75)***	1.38 (1.30, 1.46)***
***Cut point 1***	n.a.	n.a.	-0.06	n.a.
***Cut point 2***	n.a.	n.a.	0.38	n.a.
**Health region peer group_Var(Constant)**	0.071***	0.095***	0.087***	0.039***

*Note* ***p < 0.001, **p < 0.01, *p < 0.05. n.a. refers to not available

The results also show that socioeconomic status may have a protective effect on smoking behaviors of Canadians. Higher education, income, and homeownership are associated with lower odds of heavy smoking and higher odds of quitting. The association between Canadians’ social connections and their smoking behaviors is similar. Compared to married people, those who are single, widowed, divorced, or separated are more likely to smoke and less likely to quit, regardless of their living arrangements. The results also indicate that people who perceive stronger community ties have lower odds of smoking, but this is not related to their odds of quitting. The presence of chronic diseases or mood and anxiety disorders is correlated with an increased likelihood of heavy smoking, while mood or anxiety disorders are linked to a decreased likelihood of smoking cessation. Conversely, access to a healthcare provider is associated with a reduced probability of heavy smoking and an increased likelihood of smoking cessation. Lastly, smoking behaviors vary across health region peer groups.

### Social determinants of canadians’ physical activity and alcohol drinking behaviors

Table 4 demonstrates the association patterns between social determinants in examination and physical activity and drinking behaviors of young and middle-aged Canadians. People who are older, female, non-white, landed immigrants, or residents from British Columbia and Alberta tend to be less physically active than their counterparts. Similar to smoking, a higher socioeconomic status may have also lowered the risk of physical inactivity. Regarding social connection, having stronger community ties is positively associated with being physically active. However, people who live with their spouses are less likely to exercise than those who are not married, whether they live alone or not. In addition, Canadians who have chronic diseases, mood problems, or healthcare providers (possibly due to poor health) are less likely to be physically active with statistical significance.

**Table 4:** Odds ratios from two-level ordinal logistic regressions predicting physical inactivity and current drinking status, and incidence risk ratios from hierarchical negative binominal regressions predicting drinking frequency, Canadian Community Health Survey 2017–2018, N of individual size = 59,799, N of health region peer group = 8

	Physical inactivity	Drinking frequency	Drinking status
***Demographic background***			
**Age groups (18–29 years)**			
30–39 years	1.14 (1.08, 1.20)***	1.02 (1.00, 1.03)	0.88 (0.84, 0.94)***
40–49 years	1.31 (1.24, 1.38)***	1.01 (0.99, 1.03)	0.80 (0.75, 0.85)***
50–59 years	1.53 (1.45, 1.62)***	1.04 (1.02, 1.05)***	0.71 (0.67, 0.76)***
**Gender (Male)**			
Female	1.30 (1.25, 1.34)***	0.81 (0.80, 0.82)***	0.62 (0.60, 0.64)***
**Race (Non-minorities)**			
Minorities	1.22 (1.17, 1.28)***	0.76 (0.75, 0.77)***	0.51 (0.49, 0.54)***
Missing	1.32 (1.05, 1.66)*	0.91 (0.84, 0.98)*	0.69 (0.55, 0.86)**
**Immigrant status (Native-born)**			
Landed immigrant	1.49 (1.41, 1.57)***	0.77 (0.76, 0.79)***	0.49 (0.47, 0.52)***
Missing	1.28 (1.08, 1.51)**	0.90 (0.85, 0.96)**	0.66 (0.56, 0.78)***
**Province (Ontario)**			
Quebec	1.22 (1.16, 1.28)***	1.07 (1.06, 1.09)***	1.30 (1.23, 1.37)***
British Columbia	0.64 (0.60, 0.68)***	1.08 (1.06, 1.10)***	1.29 (1.21, 1.37)***
Alberta	0.91 (0.86, 0.97)**	0.96 (0.94, 0.98)***	0.94 (0.88, 1.01)
Others	1.00 (0.95, 1.05)	0.93 (0.92, 0.95)***	0.92 (0.87, 0.97)**
***Socioeconomic status***			
**Education (Primary or below)**			
Middle/High school	0.73 (0.68, 0.78)***	1.15 (1.12, 1.18)***	1.44 (1.34, 1.54)***
Some colleges or above	0.60 (0.56, 0.64)***	1.26 (1.23, 1.30)***	1.91 (1.78, 2.04)***
Missing	0.80 (0.67, 0.94)**	1.15 (1.08, 1.22)***	1.42 (1.19, 1.69)***
**Household income (< 20k)**			
20k-39.9k	0.95 (0.89, 1.02)	1.10 (1.07, 1.14)***	1.24 (1.15, 1.34)***
40k-59.9k	0.91 (0.85, 0.97)**	1.16 (1.12, 1.19)***	1.38 (1.29, 1.49)***
60k-79.9k	0.83 (0.77, 0.90)***	1.20 (1.16, 1.24)***	1.54 (1.43, 1.66)***
80k+	0.74 (0.69, 0.79)***	1.33 (1.30, 1.37)***	2.27 (2.12, 2.43)***
**Household ownership (Rent)**			
Owned	1.06 (1.02, 1.11)**	1.08 (1.06, 1.09)***	1.24 (1.19, 1.30)***
Missing	0.84 (0.64, 1.11)	1.03 (0.93, 1.14)	1.29 (0.96, 1.72)
***Social connections***			
**Marital status and living arrangements** **(Married**, **not living alone)**			
Married, living alone	1.05 (0.89, 1.23)	1.00 (0.93, 1.07)	1.02 (0.85, 1.23)
Common-law, not living alone	0.91 (0.86, 0.96)***	1.10 (1.08, 1.12)***	1.34 (1.27, 1.42)***
Common-law, living alone	0.62 (0.43, 0.89)**	1.21 (1.11, 1.33)***	1.95 (1.34, 2.82)***
Widowed/Divorced/Separated, not living alone	0.89 (0.83, 0.97)**	1.06 (1.03, 1.09)***	1.34 (1.24, 1.46)***
Widowed/Divorced/Separated, living alone	0.82 (0.77, 0.88)***	1.13 (1.11, 1.16)***	1.49 (1.38, 1.61)***
Single, not living alone	0.80 (0.76, 0.85)***	0.96 (0.94, 0.98)***	0.96 (0.91, 1.02)
Single, living alone	0.79 (0.75, 0.84)***	1.11 (1.09, 1.13)***	1.37 (1.29, 1.46)***
**Sense of belongingness (Very weak)**			
Somewhat weak	0.82 (0.77, 0.88)***	1.04 (1.02, 1.07)**	1.24 (1.15, 1.33)***
Somewhat strong	0.71 (0.67, 0.75)***	1.05 (1.02, 1.07)***	1.23 (1.15, 1.31)***
Very strong	0.65 (0.61, 0.70)***	1.05 (1.02, 1.07)**	1.11 (1.03, 1.20)**
Missing	1.56 (1.36, 1.79)***	0.74 (0.70, 0.79)***	0.49 (0.42, 0.56)***
***Health conditions***			
**Chronic conditions**			
Has at least one chronic disease	1.09 (1.05, 1.13)***	0.94 (0.93, 0.95)***	0.80 (0.77, 0.83)***
**Has mood issues (No)**			
Yes	1.10 (1.04, 1.16)**	0.96 (0.93, 0.98)***	0.87 (0.82, 0.93)***
**Has anxiety issues (No)**			
Yes	0.99 (0.94, 1.05)	0.99 (0.97, 1.01)	0.90 (0.85,0.96)***
**Has healthcare provider (No)**			
Yes	1.05 (1.00, 1.10)*	0.96 (0.95, 0.98)***	0.90 (0.86, 0.95)***
***Cut point 1***	0.00	n.a.	-1.20
***Cut point 2***	1.28	n.a.	-0.12
**Health region peer group_Var(Constant)**	0.032***	0.003***	0.014***

*Note* ***p < 0.001, **p < 0.01, *p < 0.05. n.a. refers to not available

Regarding drinking behaviors, first, people who are older, female, non-white, or landed immigrants tend to drink less often than their counterparts. People residing in Quebec and British Columbia are more prone to frequent alcohol drinking behavior compared to those in Ontario, while those in Alberta and other provinces/territories are less likely to engage in such behavior. Interestingly, the association patterns between socioeconomic factors that are related to frequent alcohol drinking are different from the patterns of the other health behaviors. Specifically, socioeconomic status, on the other hand, is not a protective factor but a risk factor for frequent alcohol drinking. People with higher education, income, and assets tend to drink more frequently and identify as regular drinkers. In terms of social connections, people who never married and live alone, as well as those who are common-law, widowed, divorced, or separated, tend to drink more heavily than those who are married and live with their spouses. However, people who perceive to have strong community ties also tend to drink more than those who have weak ties. Regarding health conditions, having chronic diseases or mood problems and having a healthcare provider are related to lower likelihoods of frequent alcohol drinking. Moreover, in the Canadian context, there are observable differences in physical inactivity and alcohol consumption across various health region peer groups. However, these differences are less pronounced compared to those observed in smoking behaviors.

### Robustness checks

We performed several tests to ensure the validity of our results. We included respondents with missing values in the physical activity variable when predicting smoking and drinking behaviors. Moreover, we used generalized ordinal logistic regressions and ordinary least squares regression (OLS) to estimate alcohol drinking frequency. In addition, we created a new variable for drinking frequency by converting the original values to the number of times of alcohol consumption per year. For instance, we coded “once per month” as “twelve times per year” and “two to three times per month” as “thirty times per year (2.5*12).” The new variable has more distinct intervals between its values. We further performed negative binomial regressions using sampling weight. The results for the main associations of interest are similar to those using the original drinking frequency variable, but the AIC and BIC values are higher, suggesting a better fit for the original variable. These tests confirmed, validated, and reinforced our main findings and strengthened their reliability.

## Discussion

This study concentrates on three health lifestyles: cigarette smoking, physical inactivity, and frequent alcohol consumption, all of which significantly impact individuals’ health. Specifically, smoking has been unequivocally established as harmful to health [33, 34]. Brenner et al. [35] have identified a correlation between smoking and elevated lung cancer rates among Canadians, noting a concurrent decline in both these rates in recent years. Similarly, alcohol consumption has been globally recognized as a health risk factor, with abstinence being the healthiest option [36]. Furthermore, a growing body of evidence underscores the role of physical inactivity in obesity, chronic conditions, and increased mortality [37–39]. The societal implications of cigarette smoking, physical inactivity, and frequent alcohol consumption are significant, as they contribute to public health challenges universally.

In this study, we utilized data from a national survey to explore the association between these three health lifestyles and the demographic characteristics, socioeconomic status, social ties, and health conditions of young and middle-aged Canadians. Our findings reveal that the relationships between the social determinants under examination and Canadians’ health lifestyles are multidirectional rather than unidirectional. These relationships encompass both positive and negative directions. Certain determinants may correlate with improved performance in some health behaviors while simultaneously correlating with poorer performance in other health behaviors. This underscores the complexity of the social determinants of health lifestyles among young and middle-aged Canadians.

Cockerham [25] indicates that individuals’ demographic features are determinants of their health lifestyles. Our first main finding concerns demographic variations in health lifestyles. Middle-aged Canadians or older ones tend to smoke and be physically inactive more, but have less frequent alcohol consumption issues. Women have lower rates of smoking and drinking than men, but also have lower levels of physical activity. Racial minorities have fewer drinking problems, but more smoking and physical inactivity issues. As expected, landed immigrants in Canada are less likely to smoke and drink, and more likely to exercise than natives. We also observed interesting geographic differences between Canadian provinces regarding residents’ health lifestyles. For instance, while residents of Quebec and British Columbia are less likely to smoke compared to those in Ontario, they tend to drink more frequently.

The second main finding focuses on the influence of socioeconomic position on people’s health lifestyles. Education, income, and household ownership are all positively related to smoking and physical activity; however, for drinking, better socioeconomic status is linked to heavier drinking. This is in line with the research conducted by Chai and Mei [40] which focused on the health behaviors of older Canadians, finding that the patterns of alcohol consumption diverged significantly from those of smoking and physical inactivity among older Canadians. Although medical evidence has demonstrated the harmfulness of alcohol consumption [35], a possible explanation for this positive association is that drinking may have offered Canadians social opportunities. Specifically, a higher socioeconomic status may imply an increased access to social environments, which in turn, could potentially escalate the consumption of alcohol. However, the significant linkage between higher socioeconomic status and higher demands for drinking can still be problem that should be taken seriously in Canada [41]. A thorough exploration of the fundamental factors contributing to higher levels of alcohol intake among Canadian adults with better socioeconomic status may equip policymakers with the necessary insights to effectively tackle these challenges. This methodology could potentially generate more accurate design of interventions and more influential public health policies, promoting healthier lifestyle choices within this demographic.

Moreover, social ties are closely related to Canadians’ health lifestyles. Our research demonstrates that marriage has a crucial role in protecting Canadians from heavy smoking and drinking behaviors, but married people are also less likely to exercise, regardless of living arrangements. The sense of belonging to their communities is a beneficial factor for all three health behaviors, as stronger ties imply healthier lifestyles. This highlights the need to pay more attention to those residents who may face social isolation issues.

We also found that both physical and mental health conditions are strongly associated with people’s health lifestyles. It is not a simple, one-directional relationship. Although people who have some specific health problems (e.g., chronic diseases, mood issues) tend to smoke more and exercise less, they also tend to drink less heavily. Moreover, importantly, there is a two-way causality that objective health issues may also affect and shape people’s health behaviors, which is not addressed by this research.

Finally, significant disparities, particularly in smoking behaviors, were noted across the eight health region peer groups as classified by Statistics Canada at the health region level. This implies that regional factors, such as socioeconomic development and the nature of the work environment, may have a considerable impact on individual health behaviors.

In summary, our study delineates the significant correlation between social determinants with respect to social factors and the health lifestyles of Canadians. It is evident that structural elements bear a close relation to behaviors such as smoking, drinking, and physical activity among Canadian adults. According to Cockerham [25], the formation of health behaviors and lifestyles is influenced by the interplay between social structures and individual agency throughout one’s socialization process. Consequently, healthy or unhealthy lifestyles can reproduce themselves under the consistent impact of these systematic factors. Our findings, therefore, do not suggest that advocating for abstinence at an individual level is the healthiest choice independent of societal norms and expectations. Instead, they underscore the potential and substantial influence of systematic factors on individuals’ health behaviors, indicating areas where policy interventions could be effective. Specifically, in our study, we identified an array of social determinants that could potentially influence individuals’ health behaviors. These influences can vary in magnitude and direction across various health lifestyles, which underscores the necessity for precise health intervention strategies that are tailored to distinct subpopulations. For instance, the frequent alcohol consumption diverges from the other two health lifestyles and exhibits a positive correlation with individuals’ socioeconomic statuses. An additional illustration is that Canadian individuals cohabiting with partners exhibit a lower propensity for frequent smoking relative to their counterparts who are widowed, divorced, or single. However, paradoxically, they also demonstrate a higher likelihood of leading physically inactive lifestyles. Viewed from this perspective, our research offers a comprehensive overview and a robust foundation for the meticulous development of policy interventions within the Canadian milieu.

### Limitations

This study acknowledges several limitations. Primarily, the research was confined to the examination of three types of health lifestyles, thus providing scope for future investigations to encompass a broader range of health lifestyles, including sleep patterns and dietary intake. Secondly, although the study incorporates factors at the health-region level, it does not sufficiently capture the neighborhood or community-level conditions experienced by the respondents. These conditions include the quality of the neighborhood, social connections, community support where the respondents reside, and so forth. These meso-level factors are crucial in shaping individuals’ health behaviors and lifestyles [24]. The implications of social interactions outside the respondents’ households on their health behaviors and lifestyles were not comprehensively addressed in our study. Lastly, the study did not delve into the factors that influence changes in Canadians’ health lifestyles, particularly those underlying factors that precipitated shifts in health lifestyles in the wake of the COVID-19 pandemic.

## Conclusions

Prior research [18, 42] has substantiated that health lifestyles significantly influence individuals’ physical and mental well-being. Consequently, discerning the social determinants of health lifestyles can equip policymakers with the necessary insights to devise effective interventions aimed at promoting healthy behaviors among specific demographic groups. Despite the limitations outlined earlier, our findings illuminate the intricate nature of the social determinants influencing individuals’ health lifestyles. This knowledge is crucial for public health action and policy formulation, enabling the implementation of tailored policies for different health behaviors. Community-level health education and interventions targeting smoking, physical inactivity, and alcohol consumption should be initiated.

In light of our findings, we propose the following targeted health interventions. Firstly, interventions aimed at reducing frequent smoking should be directed towards individuals who self-identify as male, are of an older age demographic, are native-born, belong to racial minorities, possess lower educational attainments, have lower household income levels, are divorced, widowed, or single, have weak connections with their residential community, and those who have chronic diseases or mental health issues. Secondly, interventions aimed at addressing physical inactivity should be targeted towards individuals who self-identify as female, are of an older age and are immigrants, belong to racial minorities, have lower educational and income levels, are partnered, have a weak sense of belonging to their residential community, exhibit limited social participation, and those who have chronic diseases and mood disorders. Thirdly, interventions aimed at curbing frequent alcohol consumption should be designed for individuals who self-identify as male, are younger, are non-minorities and native-born, possess higher education and superior socioeconomic positions, are not married, have strong community connections, and do not have chronic diseases or mental health problems. Lastly, it is important that associated interventions take into account the disparities among various health regions in Canada, each characterized by distinct demographic profiles and levels of socioeconomic development. These interventions should be designed with a focus on specific subgroups, taking into account their demographic and socioeconomic characteristics, social connections, and health conditions.

## Data Availability

The current study used and analyzed the public-use dataset of the Canadian Community Health Survey Annual Component for 2017-2018. The corresponding author can provide the dataset upon reasonable requests.
